# Ultra-low dose external beam radiotherapy for presumed choroidal lymphoma: a case report

**DOI:** 10.1186/s12348-022-00288-0

**Published:** 2022-03-05

**Authors:** Jeremy P. M. Flanagan, Michael Ng, Awet Z. Kibrom, Robin J. A. Filshie, Richard J. Stawell, Roderick F. O’Day

**Affiliations:** 1 Ophthalmology, Department of Surgery, University of Melbourne, Melbourne, Australia; 2 Department of Radiation Oncology, GenesisCare St Vincent’s Hospital Melbourne, Melbourne, Australia; 3 Department of Haematology, St Vincent’s Hospital, Melbourne, Australia; 4 Royal Victorian Eye and Ear Hospital, Melbourne, Australia; 5 Centre for Eye Research Australia, University of Melbourne, Melbourne, Australia

## Abstract

Primary choroidal lymphoma is a rare, slowly progressive intraocular malignancy. Most are low grade B cell lymphomas, often involving tissues adjacent to the choroid such as the subconjunctival space, lacrimal gland or orbit. Ideally, these lesions are biopsied to establish histopathological diagnosis. The most accessible ocular structure is biopsied. Obtaining tissue by transvitreal choroidal biopsy imparts a small but significant risk of ocular morbidity, including the need for multiple surgeries, retinal detachment and vision loss.

External beam radiotherapy (EBRT) is a common and effective treatment of low-grade lymphomas. EBRT has been found to very successfully treat primary marginal zone lymphomas of the ocular adnexa, which are typically of the same cell type as most primary choroid lymphomas. Ultra-low dose EBRT, most commonly using a total dose of 4 Gy, has been shown to be as effective as higher doses of radiotherapy for follicular or marginal zone lymphomas. The use of this low dose regimen for conjunctival lymphomas has been recently explored. The role of EBRT, and especially ultra-low dose EBRT, for treatment of primary choroidal lymphoma has been confined to case reports.

We describe a case of presumed primary choroidal lymphoma diagnosed on clinical findings alone as the risks of ocular biopsy were deemed too high, and report outcome following treatment with ultra-low dose EBRT.

## Introduction

Primary choroidal lymphomas are rare malignancies that are often unilateral, without systemic involvement and of low-grade B cell type [1, 2]. They usually present in patients over 60 years old with slowly-progressive unilateral vision loss, but may also be associated with eye pain or elevated intraocular pressure, or be entirely asymptomatic [1, 3]. Assessment of a biopsy specimen is used to establish the diagnosis, facilitate histological subtyping and direct treatment of these lesions [3]. When these lesions are confined to the choroid without involvement of the ocular surface, adnexa or orbit [3], lesion biopsy is performed by way of transvitreal fine-needle aspiration (FNA). Transvitreal FNA carries a risk of iatrogenic complications, including retinal detachment, cataract and infection, and the potential for insufficient tissue sampling for diagnosis [1, 4]. Transvitreal FNA biopsy rates of choroid tumours with cytologic non-diagnosis remains persistently high [5, 6], with up to half of all biopsies generating insufficient aspirates in thinner choroidal lesions [7–10].

There is no standardised treatment for primary choroidal lymphoma due to its rarity, however external beam radiotherapy (EBRT) has been shown to be an effective treatment with median doses of 30–36 Gy in 1.8–2Gy daily fractions [1, 3]. Common complications at this radiation dose include dry eye, cataract and retinopathy [3, 11]. Ultra-low dose radiotherapy (4 Gy dose delivered in 2 × 2 Gy fractions) has been found to successfully treat primary ocular adnexal lymphomas whilst minimizing side-effects [12–14]. This regimen has also been reported in patients with primary choroidal lymphoma recently [15–18]. However, in each of these recent case reports, diagnosis was confirmed by tissue biopsy. Although the risk of a significant complication is relatively low, patient and disease factors such as small volume disease and preserved visual function, may align such that treatment without tissue diagnosis is the preferred management plan. We describe a successful case of ultra-low dose EBRT for presumed choroidal lymphoma without tissue diagnosis.

## Case report

A 73-year-old male with an ophthalmic history including ocular hypertension, allergic conjunctivitis, early cataracts and a family history of glaucoma was referred with unilateral, progressive choroidal infiltrate in his right eye 18 months after being found to have unilateral choroidal folds in the same eye. Aside from being a former smoker, past medical history was otherwise unremarkable and the patient denied any constitutional or B symptoms.

**Fig. 1:**
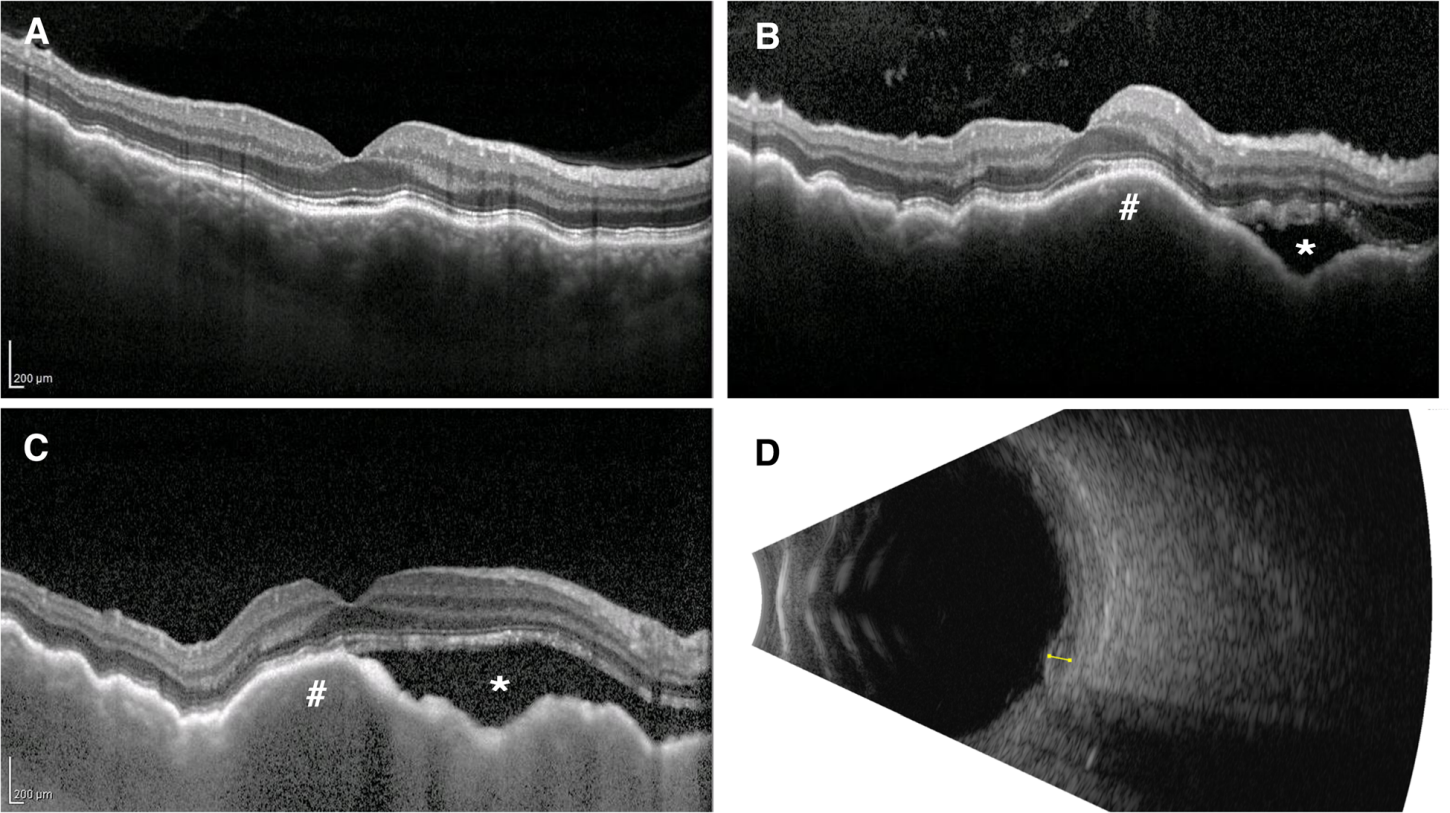
EDI-OCT of patient prior to treatment initiation. **a**) EDI-OCT at initial diagnosis of choroid folds; **b**) EDI-OCT following worsening of symptoms 11 months later; **c**) EDI-OCT on presentation to ophthalmology oncology clinic; **d**) ocular ultrasound with yellow line showing lesion depth of 1.5 mm. EDI-OCT = enhanced depth imaging optical coherence tomography’ * = subretinal fluid; # = choroidal lesion

**Fig. 2:**
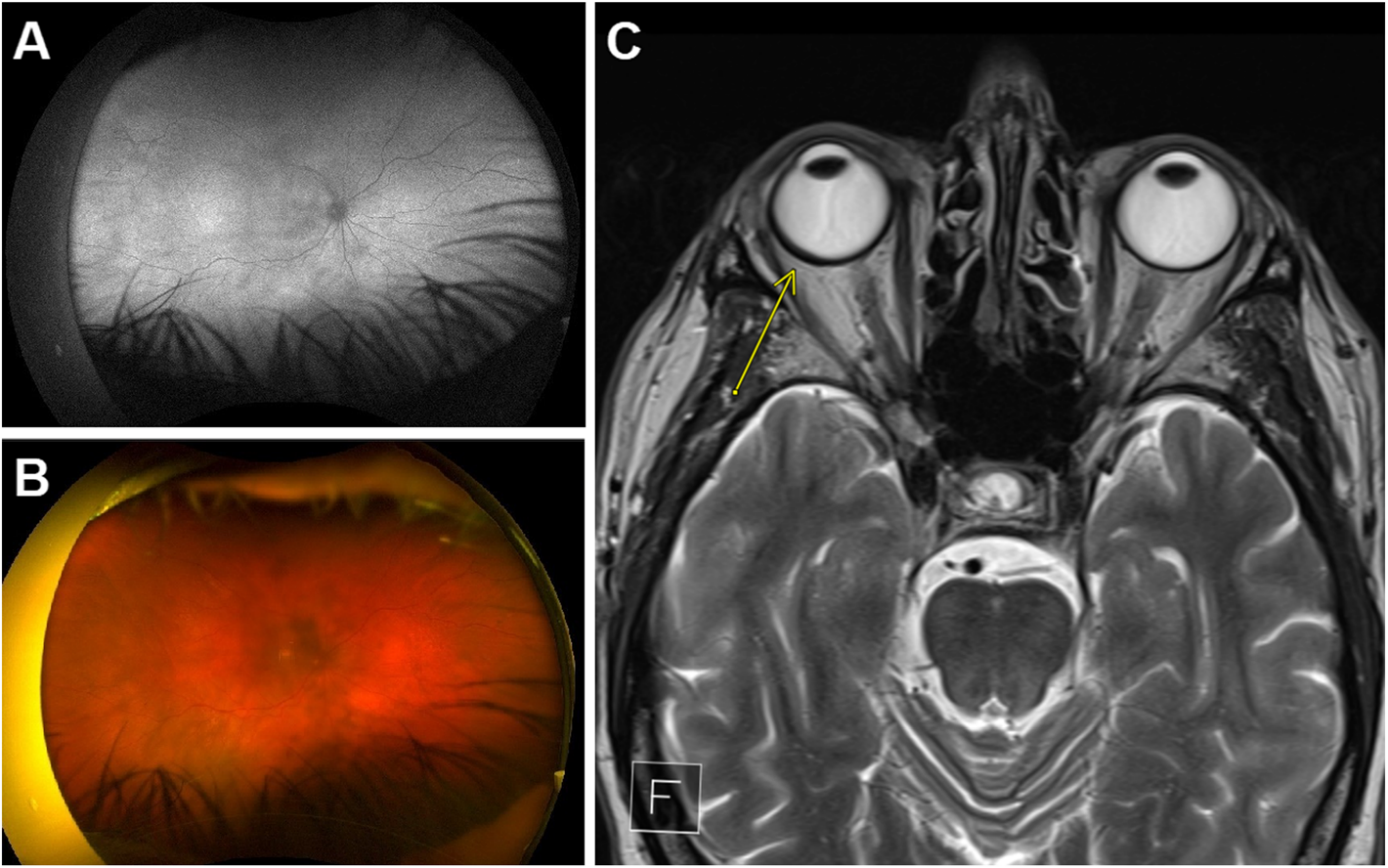
Imaging prior to treatment initiation. Right eye **a**) fundus autofluorescence and **b**) pseudocolour images on presentation to ophthalmology oncology clinic; **c**) transverse orbital T2-weighted MRI image taken as part of systemic workup, with yellow arrow highlighting asymmetric thickening and enhancement of the wall of the right globe

The patient was initially diagnosed with unilateral choroidal folds in his right eye by his ophthalmologist via enhanced-depth imaging optical coherence tomography (EDI-OCT; Heidelberg Spectralis, Heidelberg Engineering, Heidelberg, Germany; Fig. 1a), with right eye best corrected visual acuity (BCVA) 20/20 and left eye 20/16. After remaining stable for 11 months, right eye BCVA rapidly declined over a three-month period to 20/60, due to progressive cataract and choroidal infiltrate. There was no clinical evidence of conjunctival, ocular adnexal or orbital involvement in either eye. There was no intraocular inflammation. A posterior subcapsular cataract had developed in the right eye and a pale choroidal infiltrate was now evident on slit-lamp examination, with increased choroidal thickening on EDI-OCT and overlying subretinal fluid (Fig. 1b, c). Right eye ocular ultrasound showed diffuse, hyperechoic choroidal thickening (Fig. 1d), no abnormality was apparent on right eye fundus autofluorescence imaging (Fig. 2a), and right eye pseudocolour imaging showed a hazy view of the fundus (Fig. 2b). A trial of oral high dose prednisolone (75 mg) daily had no effect and was tapered after a month of no improvement in right BCVA or choroidal disease. The clinical and imaging features were highly suggestive of primary choroidal lymphoma. Vitreoretinal surgery review concluded that the potential ocular morbidity of a transvitreal choroidal biopsy were too high given the patient’s relatively good vision and potentially low diagnostic yield.

Clinical review and systemic workup (full body positron emission tomography (PET), lumbar puncture, brain Magnetic Resonance Imaging (MRI) and bone marrow biopsy) by the haematologist did not detect locoregional or systemic disease, with brain MRI showing only a mild unilateral enhancement and thickening of the lateral wall of the right globe (Fig. 2c) and bone marrow biopsy showing low counts of two B cell clones in the bone marrow, the first being indicative of monoclonal B lymphocytosis and the second consistent with lymphoma.

A multidisciplinary team review, consisting of representatives from ophthalmology, haematology and radiation oncology, discussed management and it was decided to treat this patient with ultra-low dose EBRT for presumed right primary choroidal lymphoma without histological diagnosis. A total dose of 4 Gy to be delivered in 2 fractions on consecutive days to the entire orbit was recommended.

To ensure precise positioning, a radiotherapy immobilisation mask (Klarity Green® Thermoplastic S-Type mask) was used for computerised tomography (CT) simulation and planning. The patient was positioned supine on the CT scanner couch, with anatomical landmarks and positioning lasers used to ensure patient and mask alignment. The CT scan was performed (Siemens Somatom Definition AS CT Scanner (Siemens AG, Munich, Germany)) with scan slice thickness of 2 mm.

**Fig. 3:**
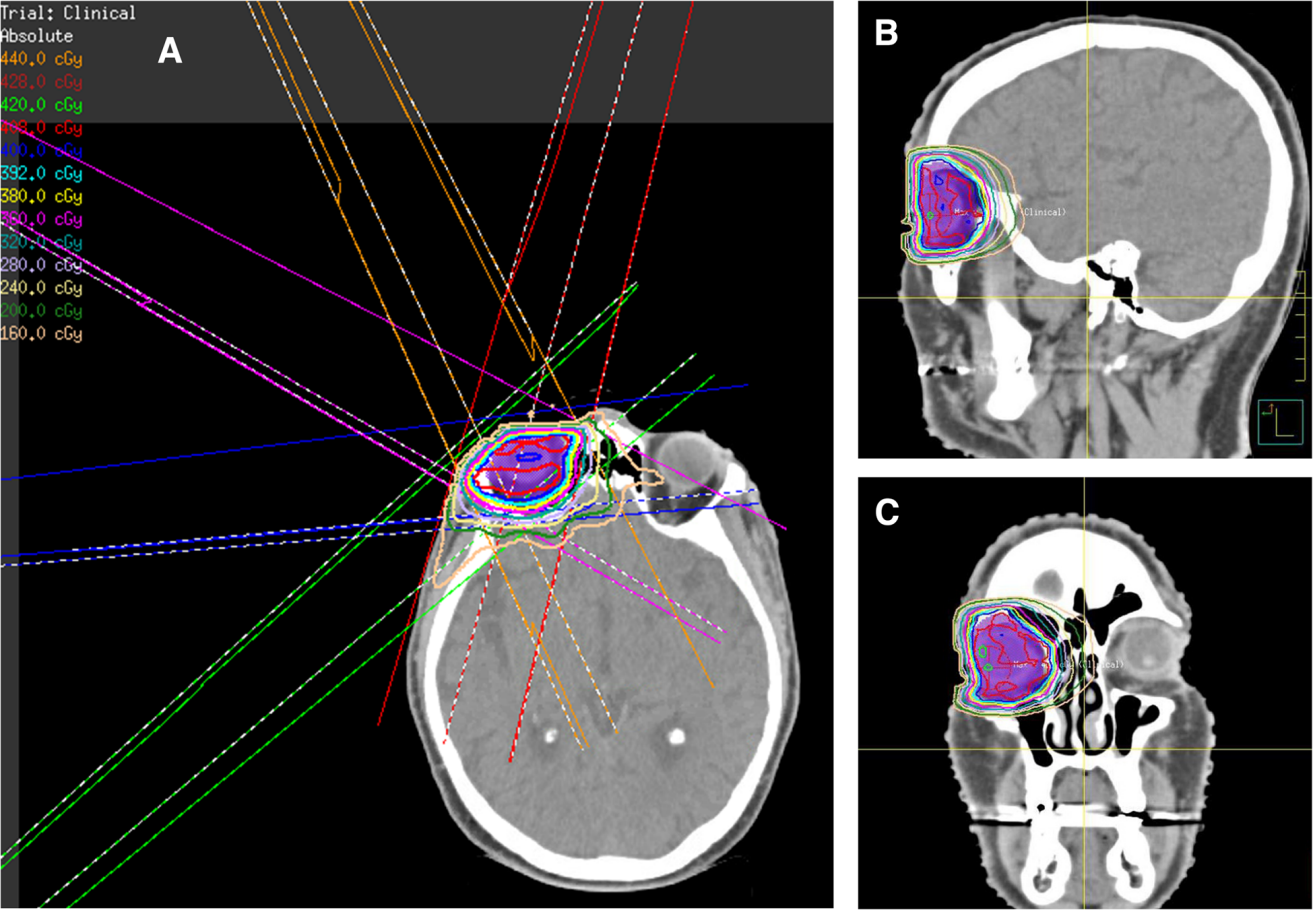
Dose delivery and IMRT Beam Arrangement at 15°, 230°, 265°, 300°, 335°: **a**) axial view; **b**) dose delivery, lateral view; **c**) dose delivery, coronal view. IMRT = intensity-modulated radiation therapy

Target voluming on CT was performed using MIM Maestro® Version 6.8.9 (MIM Software Inc., Beachwood, OH, US). The clinical target volume (CTV) was the entire orbit, with a margin expansion of 3 mm to create the planning target volume (PTV). Pinnacle Treatment Planning System Version 3 (Philips, Fitchburg, WI, USA) was used to create an Intensity-modulated Radiation Therapy (IMRT) plan. The IMRT plan consisted of a 5-beam arrangement at gantry angles of 15, 230, 265, 300 and 335 degrees, using 6MV energy photons as shown in Fig. 3. These beam arrangements were chosen to ensure conformal target coverage of the PTV and minimise exit dose to contralateral orbit. The plan was optimised to ICRU 83 guidelines [19] with the PTV receiving a minimum of 97.5% of the dose and a point maximum dose of 106.1%, to a prescribed dose of 4 Gy in 2 fractions on consecutive days.

Treatment was commenced 1 day following simulation scan and delivered over consecutive days on the Elekta Versa HD linear accelerator with Agility Multi-Leaf Collimator (MLC). Image guidance utilized pre-treatment cone-beam computed tomography (CBCT) to ensure accurate delivery of radiation to the orbit. No side effects were reported during treatment.

**Fig. 4:**
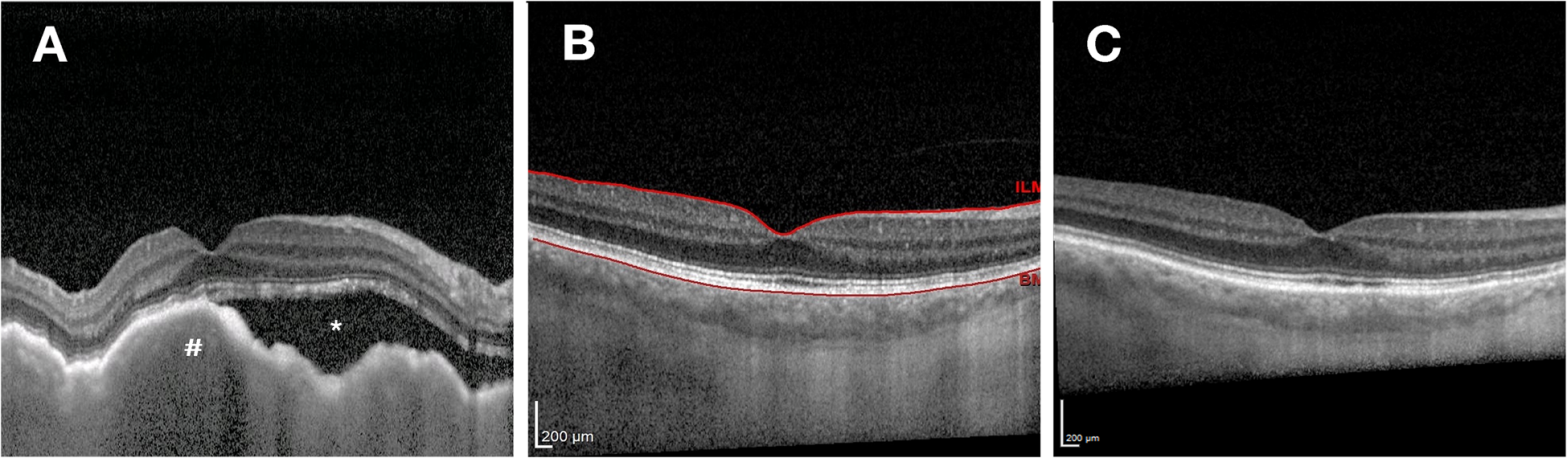
EDI-OCT of right eye. (**a**) prior to radiotherapy; and **b**) 6 months following ultra-low dose EBRT and cataract surgery **c**) 12 months following ultra-low dose EBRT and cataract surgery. EDI-OCT = enhanced depth imaging optical coherence tomography; EBRT = External Beam Radiotherapy; * = subretinal fluid; # = choroidal lesion

Follow up 1 month after radiotherapy treatment revealed resolution of choroidal infiltrate on EDI-OCT, with right eye vision remaining reduced at 20/80 largely due to the posterior subcapsular cataract. Following right-sided cataract surgery 6 months later, right eye BCVA improved to 20/16. There was no clinical or EDI-OCT evidence of disease recurrence at 6 or 12 months follow up (Fig. 4b, c) and right eye BCVA was stable at 20/16.

## Discussion

Primary indolent B cell lymphomas of the eye most frequently involve the ocular adnexa – the conjunctiva, lacrimal gland or orbit. Primary lesions solely involving the choroid are extremely rare. Accordingly, much of the literature regarding treatment of these lesions is with reference to the ocular adnexa, rather than the uveal tract.

Indolent B cell lymphomas are particularly sensitive to radiotherapy. Moderate-dose EBRT (20–30 Gy delivered in 1.8–2 Gy daily fractions) has been the gold-standard treatment for the last number of decades. B cell lymphomas of the ocular system have traditionally been treated with similar doses with consistently high rates of local control for lesions of the adnexa [20, 21]. Treatment of lesions of the choroid with similar EBRT doses (mean 30–36 Gy in 1.8–2 Gy daily fractions) has generated similar rates of tumour regression and local control, albeit with iatrogenic cataract, dry eye syndrome and decrease in BCVA being a common consequence of radiotherapy [1, 3]. However, there has been increasing interest to reduce potential toxicity of lymphoma radiotherapy using lower doses. One randomized trial found no difference in disease response with standard dose (40–45 Gy) versus lower dose (24 Gy) for indolent systemic lymphomas [22] and another randomized trial in follicular and marginal zone lymphoma comparing 24 Gy versus 4 Gy doses showed slightly inferior local control with lower dose but less toxicity [23].

Ultra-low dose radiotherapy is an appropriate second line treatment option for lymphomas of the ocular system, given the desire to reduce potential long term radiotherapy complications, the unique radiosensitivity of these tumours and the ability to use re-irradiation with higher doses as a salvage option. Ultra-low dose EBRT has shown high rates of local control with lower rates of radiotherapy-induced complications in adnexal lesions [13, 24, 25], with 24–25 Gy reirradiation as an effective secondary intervention if tumours do not respond or progress following initial treatment [12]. In contrast, ultra-low dose radiotherapy for indolent B cell lymphomas of the choroid has been described only in a few recent case reports [15–18]. Details of these six cases are presented in Table 1 alongside our case for comparison.

**Table 1:** Primary choroidal lymphomas treated with 2 x 2 Gy ultra-low-dose radiotherapy

Publication	Age	Eye	BCVA of affected eye	Tumour depth at time of biopsy (mm)	Biopsy method	Follow up (months)	BCVA at follow up
Shields et al. [15]	67	Right	20/50	Unknown	FNA (adnexa)	24	20/30
Yang et al. [16]	64	Left	20/40	2.9	Unknown (adnexa)	6	20/25
	74	Left	20/50	1.9	FNA	11	20/40
	72	Right	20/50	4.0	FNA	24	20/25
Dirani et al. [17]	89	Right	Hand motion	Unknown	23-gauge PPV	6	Count fingers
Kam et al. [18]	72	Right	Hand motion	7	27-gauge PPV	3	Count fingers
Our case	73	Right	20/60 (with cataract)	1.5	NONE	10	20/16 (post-CSx)

*BCVA* best corrected visual acuity, *FNA* Fine needle aspiration, *PPV* pars-plana vitrectomy, *CSx* cataract surgery

Patients in these prior reports had poor BCVA in their affected eye, at 20/40 (1 case) 20/50 (3 cases) and only hand movements (2 cases). In contrast, our patient had relatively preserved vision at 20/60 and cataract consistent with this level of vision. Lesions in these cases were 2.9 mm, 1.9 mm, 4 mm and 7 mm in depth, with two studies not stating lesion depth at time of biopsy. In one case where disease was not responsive to initial ultra-low dose EBRT at 5 weeks post-treatment, follow up treatment with 24 Gy in 12 fractions was delivered and associated with the resolution of both subretinal fluid and choroidal thickening [18].

In all prior cases diagnostic lesion biopsy was performed. Two were taken from involved structures located anterior to the choroid and four were transvitreal biopsies from the choroidal lesion by FNA, with or without pars-plana vitrectomy (PPV), with minimal side effects reported. Although transvitreal choroidal biopsy is considered relatively safe with low risks of complications, it can be complicated by non-clearing vitreous hemorrhage requiring further surgery [9], cataract, retinal detachment and decreased BCVA at long-term follow up [26].

Our patient has a complete clinical response following a convenient two-day course of ultra-low dose radiotherapy, as early as 1 month following treatment without acute toxicity. Our treating team determined that ocular morbidity risk following chorioretinal biopsy outweighed potential diagnostic and treatment benefits. This took into account the high clinical suspicion that the diagnosis was primary choroidal lymphoma, the relatively preserved vision of the patient, a lack of other available biopsy sites (no adnexal involvement) and the low lesion thickness (1.5 mm) prior to treatment initiation. Although a recent study has demonstrated high diagnostic efficacy of FNA biopsy for smaller uveal tumours, the tumours in this study were still substantially thicker than the 1.5 mm lesion seen in our patient [27]. In our patient, choroidal infiltrate resolved rapidly following radiotherapy and has not recurred to date, and vision improved to 6/5 12 months after radiotherapy treatment and cataract surgery.

In conclusion, tissue diagnosis of ocular lesions is important to accurately identify lesion identity and dictate an appropriate course of treatment. The accepted standard of care for choroidal lymphoma remains EBRT of 20–30 Gy in consecutive 1.8–2 Gy daily fractions. However, as we have presented in this case, in certain circumstances it may be better to treat complex patients with an ultra-low regimen dose based on presumptive diagnosis.

## Data Availability

Data sharing is not applicable to this study as no datasets were generated or analysed during the current study.

## Abbreviations

EBRT: External beam radiotherapy
FNA: Fine-needle aspiration
EDI-OCT: Enhanced-depth imaging optical coherence tomography
BCVA: Best corrected visual acuity
PET: Positron emission tomography
MRI: Magnetic resonance imaging
CT: Computerized tomography
CTV: Clinical target volume
IMRT: Intensity-modulated radiation therapy
MLC: Multi-Leaf Collimator
CBCT: Cone-beam computed tomography
